# Imbalance of Serum Bone-Metabolism-Related Factors Associated with Osteonecrosis of the Jaw

**DOI:** 10.3390/biomedicines13102410

**Published:** 2025-10-01

**Authors:** Kazuyuki Yusa, Yuji Takeda, Nobuyuki Sasahara, Tomoharu Hemmi, Shigeo Ishikawa, Tsuneo Konta

**Affiliations:** 1 Department of Dentistry, Oral and Maxillofacial-Plastic and Reconstructive Surgery, Faculty of Medicine, Yamagata University, Yamagata 990-9585, Japan; nsasahara@med.id.yamagata-u.ac.jp (N.S.); henmi@med.id.yamagata-u.ac.jp (T.H.); shigeo_ishikawa2011@yahoo.co.jp (S.I.); 2 Department of Immunology, Faculty of Medicine, Yamagata University, Yamagata 990-9585, Japan; yu-takeda@med.id.yamagata-u.ac.jp; 3 Department of Public Health and Hygiene, Faculty of Medicine, Yamagata University, Yamagata 990-9585, Japan; kkonta@med.id.yamagata-u.ac.jp

**Keywords:** medication-related osteonecrosis of the jaw (MRONJ), diagnosis, bone metabolism, inflammation, bone-modifying agents (BMAs), bisphosphonates (BPs), denosumab (Dmab)

## Abstract

**Background:** Medication-related osteonecrosis of the jaw (MRONJ) is a serious adverse effect of bone-modifying agents. The aim of this study was to elucidate the pathogenesis of MRONJ through a comprehensive comparison of bone-metabolism-related factors in sera from patients with MRONJ and healthy controls. **Methods:** This study was a retrospective cross-sectional biobank analysis in which 31 patients in a non-MRONJ group and 10 patients in an MRONJ group were screened. Serum levels of 13 proteins (i.e., hormones, growth factors, and cytokines) related to bone metabolism were measured by simultaneous multi-parameter analysis using bead-based immunoassays. **Results:** The MRONJ group displayed suppressed bone metabolism with a background of chronic inflammation. In addition, a significant decrease in the expression of alkaline phosphatase liver/bone/kidney (*p* < 0.05, effect size of 0.46 (95% CI: 0.08 to 0.73)) and a significant increase (*p* < 0.05, effect size was −0.42 (95%CI: −0.72 to 0.01)) in the expression of tumor necrosis factor α were observed in the MRONJ group. **Conclusions:** These results may contribute to a better understanding of the etiology, pathophysiology, and progression of MRONJ.

## 1. Introduction

Bone-modifying agents (BMAs), such as bisphosphonates and denosumab (Dmab), have been widely used in the treatment of various conditions, including osteoporosis, bone metastases of malignant tumors and bone-destructive diseases [1,2,3,4]. Despite the many therapeutic benefits of BMAs, medication-related osteonecrosis of the jaw (MRONJ) is a serious adverse effect, and the number of cases identified as involving MRONJ has been gradually increasing. Although the pathogenesis of MRONJ remains unclear, some reports have indicated that excessive suppression of bone remodeling by BMAs may contribute to the development of MRONJ [5,6]. Based on these findings, several investigations have attempted to identify markers involved in bone metabolism in order to elucidate the pathogenesis and help predict the prognosis of MRONJ. One notable study by Marx et al. investigated whether serum levels of C-terminal telopeptide (CTX) could serve as a risk factor for the development of MRONJ [7]. That study proved particularly important because specific reference values were provided. However, when comparing groups with and without osteonecrosis of the jaw, Kwon et al. reported that although serum CTX levels tended to be slightly higher in the control group, the difference was not significant [8]. Several other studies have attempted to find biomarkers for MRONJ [9,10,11,12], but more comprehensive research is still needed. To the best of our knowledge, no clinical studies have undertaken comprehensive measurements of both bone formation and bone resorption factors using serum from MRONJ patients. In the context of MRONJ pathogenesis, suppression of bone remodeling may be associated with inflammatory inhibition—characterized by elevated TNF-α expression—and with impaired osteoblast differentiation, as suggested by the downregulation of markers such as ALPL. However, these mechanisms remain to be fully elucidated. The aim of this study was therefore to comprehensively analyze bone-metabolism-related factors in the serum of patients with MRONJ and healthy controls to elucidate the pathogenesis of MRONJ using bead-based immunoassays.

## 2. Materials and Methods

### 2.1. Data Source

This study was conducted using data from patients enrolled in the Yamagata Biobank from 2018 to 2023. Blood samples were collected who had received an explanation regarding the research use of their samples and provided informed consent. Approval for this study was obtained from the ethics committee at Yamagata University Faculty of Medicine (protocol no. 2024-227). All protocols complied with the Declaration of Helsinki and its later amendments. Information regarding the study, including details of the research and the option to decline participation based on the opt-out method, is available on our hospital website.

### 2.2. Patients and Study Design

Patients identified from the Yamagata Biobank database with a diagnosis of osteoporosis, bone metastases, osteonecrosis of the jaw, or osteomyelitis of the jaw were considered potential candidates for inclusion in this study. Among these, patients who had no history of BMA administration at the time of blood collection were excluded. The remaining patients were then categorized based on the presence or absence of MRONJ.

The diagnostic criteria for MRONJ were defined as follows: (1) a history of treatment with bisphosphonates or Dmab; (2) bone exposure in the oral, maxillofacial, or facial region persisting for at least 8 weeks; and (3) no history of radiation therapy to the jaw and absence of primary cancer or metastatic cancer involving the jawbone.

In the non-MRONJ group, patients with acute inflammation at the time of blood collection or a history of diseases affecting bone metabolism (e.g., rheumatoid arthritis, Crohn’s disease, ulcerative colitis, or renal failure) were excluded after adjusting for age. To ensure consistency in age distribution, since all participants in the MRONJ group were 75 years of age or older, individuals under the age of 75 were excluded from the non-MRONJ group. The medical history of bone-metabolism-related diseases was obtained from the patients’ medical records. Patients showing evidence of acute inflammation—defined by blood test indicators such as CRP ≥ 0.14 and WBC ≥ 8600, or by radiographic findings on X-ray or CT—were excluded from both groups in the analysis.

### 2.3. Bead-Based Immunoassay Using Flow Cytometry

Factors related to bone metabolism in serum samples obtained from study participants were measured using the LEGENDplex™ Human Metabolism Panel V02 (BioLegend, San Diego, CA, USA) in accordance with the instructions from the manufacturer. All samples were analyzed using flow cytometry (FACSMelody; BD Biosciences, San Jose, CA, USA). Data analysis was conducted using LEGENDplex™ Data Analysis Software version 8.0 (BioLegend).

### 2.4. Statistical Analysis

All statistical analyses were performed using EZR (Saitama Medical Center, Jichi Medical University, Saitama, Japan), which is a modified version of R Commander used as a graphical interface for R (The R Foundation for Statistical Computing, Vienna, Austria) [13].

## 3. Results

### 3.1. Clinical Characteristics of Patients in the Non-MRONJ and MRONJ Groups

**Figure 1:**
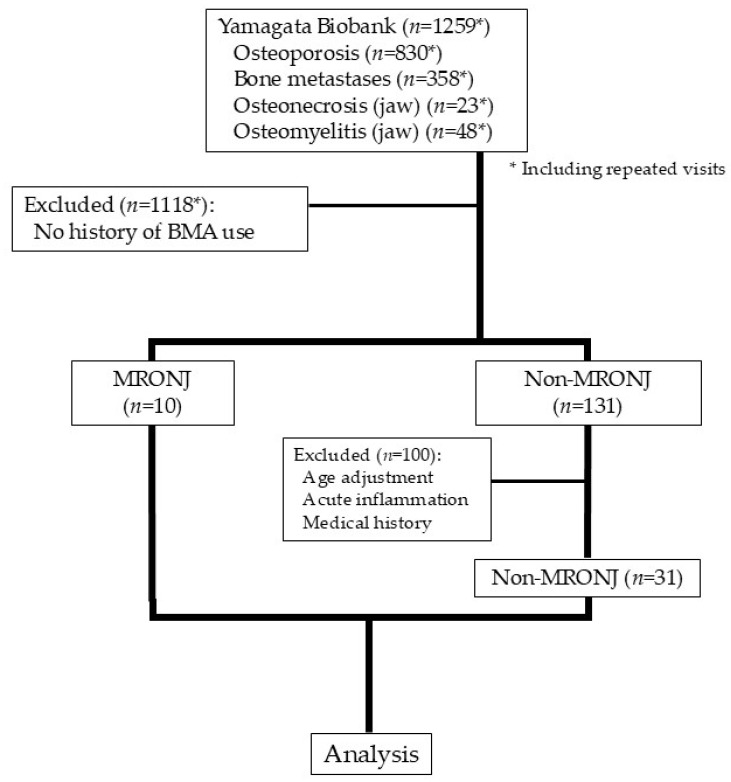
Flowchart for the selection of study participants.

A total of 1259 patients were screened for inclusion in the present study (Figure 1). Of these, 31 in the non-MRONJ group and 10 patients in the MRONJ group were included for analysis. Both groups were predominantly female, and the MRONJ group tended to include more patients receiving BMAs for bone metastasis. As for the actual BMAs administered, Dmab was the most commonly administered agent in the MRONJ group, and the route of administration was predominantly intravenous or subcutaneous in the MRONJ group. Among patients in the MRONJ group, the osteonecrosis sites were the mandible in seven cases, the maxilla in two cases, and both the mandible and the maxilla in one case. The MRONJ grade was stage II in seven cases and stage III in three cases (Table 1).

### 3.2. Inter-Correlation of Bone Metabolism Factors Associated with Osteonecrosis of the Jaw

This study measured levels of the following 13 proteins involved in bone metabolism: osteoprotegerin (OPG); osteopontin (OPN); platelet-derived growth factor-BB (PDGF-BB); alkaline phosphatase liver/bone/kidney (ALPL); acid phosphatase 5 tartrate-resistant (ACP5); leptin; receptor activator of nuclear factor-κB ligand (RANKL); tumor necrosis factor α (TNF-α); interleukin (IL)-6; parathyroid hormone (PTH); IL-1β; bone morphogenetic protein 2 (BMP-2); and Dickkopf WNT signaling pathway inhibitor 1 (DKK-1). Among these, IL-6, PTH, IL-1β, BMP-2, and DKK-1 were excluded from subsequent multivariate analysis because many samples were below the limit of detection.

A correlation matrix was constructed and factor analysis and principal component analysis (PCA) were conducted to explore the relationships among expression levels of bone-metabolism-related markers in the non-MRONJ and MRONJ groups. The KMO measure of overall MSA was 0.58, and several markers have MSA values below 0.60. While several markers may not be ideally suited for factor analysis, we decided not to exclude them because the primary objective of the factor analysis is to capture the general trends in the data, rather than achieving perfect fit. And, Bartlett’s test was significant (χ2 = 89.02, *p* = 2.20 × 10^−6^).

**Figure 2:**
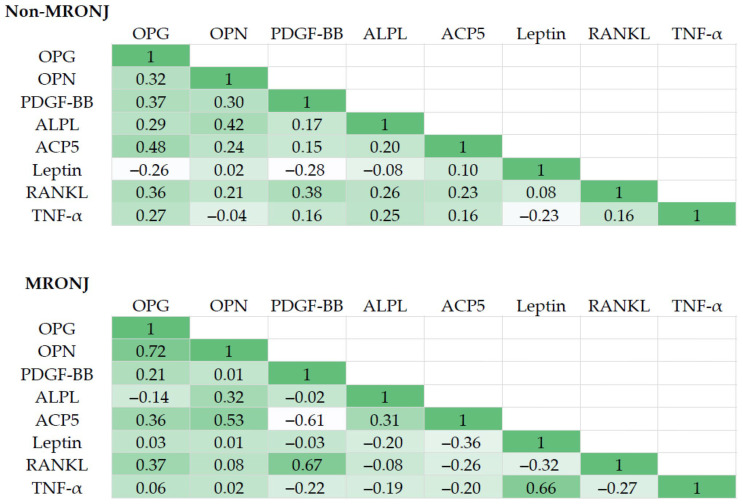
Heat map of correlation matrices for measured factors in the non-MRONJ and MRONJ groups. The number in each cell indicates the correlation coefficient (*r*). The green density indicates the magnitude of the numeric value from the max 1 to the minimum value in each matrix.

Heatmaps of the correlation matrices for representative factors in the non-MRONJ and MRONJ groups are presented in Figure 2. In the MRONJ group, a correlation was observed between TNF-α and leptin, whereas no such associations were found in the non-MRONJ group. The correlation between leptin and TNF-α is known to be associated with inflammatory diseases such as sepsis, psoriasis, preeclampsia, and obesity [14,15,16,17]. These observations suggest that chronic inflammation underlies the pathogenesis of MRONJ.

The results of factor analyses are presented in Table 2. In the MRONJ group, “Factor 1” showed high loading for OPG, OPN, and ACP5, indicating that this factor is strongly associated with bone-metabolism-related processes (comprising both bone resorption and bone formation). Although the non-MRONJ group also showed involvement of bone metabolism, the composition differed slightly from that of the MRONJ group. Moreover, “Factor 2” revealed a strong association with inflammatory factors such as TNF-α and leptin in the MRONJ group, whereas the non-MRONJ group showed no inflammation-related factors to any comparable extent.

**Figure 3:**
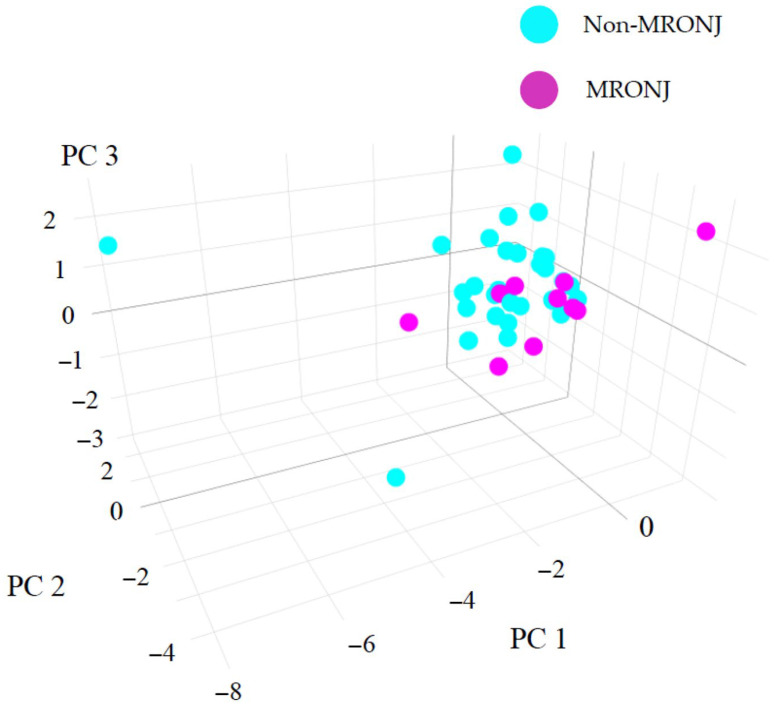
Variations in factors associated with bone metabolism obtained from the non-MRONJ (*n* = 31) and MRONJ (*n* = 10) groups presented as a three-dimensional plot using principal scores. Each principal score is plotted along the PC1, PC2, and PC2 axes. Proportions of variance for PC1, PC2, and PC3 are calculated as shown in Table 3.

Factors and their contributions were analyzed using PCA (Table 3), and an overview of the entire data set was obtained in a three-dimensional plot using principal scores (Figure 3). According to the scree plot, the variances explained by each component were as follows: PC1 = 2.84, PC2 = 1.23, PC3 = 1.12, PC4 = 0.92, PC5 = 0.67, and PC6 = 0.62, with PC1 accounting for the largest proportion of variance. The results showed no clear separation in data between the non-MRONJ and MRONJ groups. This result is at least partly consistent with observations from previous studies. However, focusing on component elements of the principal component, OPG, ALPL and ACP5 appeared to strongly influence the PC1 axis. Further, PDGF-BB, leptin and TNF-α influenced the PC2 axis. PC1 was thus related to bone metabolism and PC2 to inflammation, suggesting that inflammation is linked to the background suppression of bone metabolism.

### 3.3. Alterations in Characteristic Bone Metabolic Factors Associated with Osteonecrosis of the Jaw

**Figure 4:**
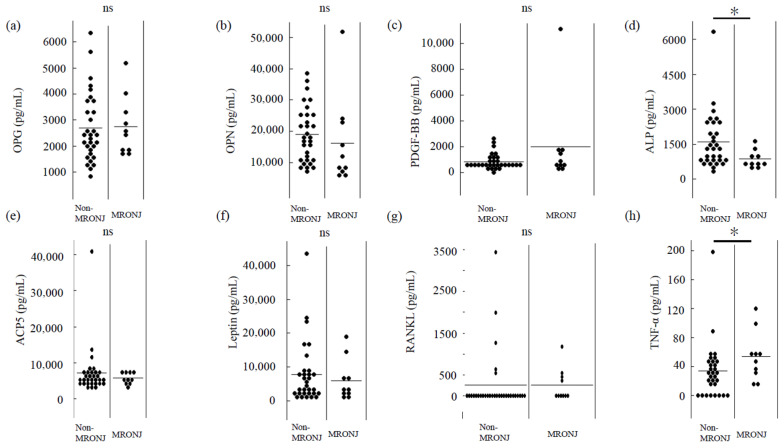
Expression of bone-metabolism-related factors in the non-MRONJ and MRONJ groups. Dot plots illustrate representative factors for the non-MRONJ and MRONJ groups (**a**) OPG, (**b**) OPN, (**c**) PDGF-BB, (**d**) ALP, (**e**) ACP5, (**f**) Leptin, (**g**) RANKL, (**h**) TNF-α. Each bar in the dot plots represents the mean value. Statistical significance was assessed using the Mann–Whitney U test. ns; non-significant * *p* < 0.05.

We examined whether the identified factors reflected the difference between non-MRONJ and MRONJ groups. As shown in Figure 4, ALPL levels were significantly lower and TNF-α levels were significantly higher in the MRONJ group. The group difference in ALPL was evaluated using Cliff’s delta, yielding an effect size of 0.46 (95% CI: 0.08 to 0.73), indicating a medium effect. For TNF-α, the effect size was −0.42 (95%CI: −0.72 to 0.01), also suggesting a medium effect.

These observations imply a potential involvement of suppressed bone formation and a heightened inflammatory milieu in MRONJ development.

## 4. Discussion

This study investigated the expression of bone-metabolism-related factors in the serum of patients in non-MRONJ and MRONJ groups. MRONJ is a benign disease of the jaw that is most often seen after oral and maxillofacial surgery, and more commonly appears among patients with malignant tumors who are receiving long-term intravenous or subcutaneous administration of zoledronic acid [18]. In recent years, an increasing number of reports have described MRONJ caused by Dmab administered for bone metastases and other conditions. While some reports have suggested that Dmab and zoledronic acid are similar in terms of the risk of MRONJ [19,20], others have reported that a higher risk is associated with Dmab use [21,22].

In the present study, intravenous or subcutaneous administration was the most common route used for BMAs in the MRONJ group. Furthermore, osteoporosis was the most common indication for BMA use, and the most frequently administered BMA in the MRONJ group was Dmab, which was used in five cases (Table 1).

These results may be influenced by the fact that this was a retrospective study designed based on database searches, and further investigations including a prospective methodology are warranted.

Treatment strategies for MRONJ include conservative and surgical therapies, and scattered studies have focused on the cure rate [23,24,25]. Notably, extensive surgical interventions involving the removal of surrounding healthy bone have been shown to be associated with higher healing rates compared to conservative surgery removing only necrotic bone [23,24,26,27]. However, concluding that extensive surgery is to be recommended based on these results is premature, because this issue needs to be discussed not only in terms of cure rate, but also in terms of postoperative oral function and quality of life for patients. Taking into account these considerations, effective treatments for MRONJ appear to remain lacking. Preventive intervention and early treatment of MRONJ are therefore important, and studies aimed at predicting the onset of MRONJ and elucidating the mechanisms of pathogenesis are needed [28,29,30]. The identification of suitable markers in serum should facilitate such goals [31,32,33,34].

As mentioned earlier, many studies have targeted CTX, but results have varied from study to study, and whether CTX can be used to predict MRONJ and is involved in its pathogenesis remain unclear [12,31,35,36]. Other parameters, such as 25-hydroxyvitamin D, osteocalcin, type 1 procollagen N-terminal propeptide, and PTH, have also been investigated, but further studies are needed to determine the correlations with MRONJ onset, progression and/or treatment response [12,36,37,38]. Therefore, an exhaustive search for biomarkers is needed in the future, and this study conducted a comprehensive analysis of bone-metabolism-related factors using bead-based immunoassay. The results of the correlation matrix, factor analysis, and PCA are shown in Table 2 and Table 3 and Figure 2 and Figure 3. The MRONJ group was assumed to have suppressed bone metabolism with a background of chronic inflammation. In addition, a significant decrease in the expression of ALPL and a significant increase in the expression of TNF-α were observed in the MRONJ group. TNF-α can regulate the expression of molecules related to inflammation, oxidative stress, apoptosis, and autophagy by activating the NF-κB signaling pathway, thereby influencing the survival and death of various cell types [39]. From the perspective of increased TNF-α expression and bone pathology, studies on avascular necrosis of the femoral head (ANFH) are particularly notable. Significant elevations in TNF-α levels have been observed in bone tissue from patients with ANFH, and these effects are reported to be mediated via the p38 mitogen-activated protein kinase (MAPK) and nuclear factor-κB (NF-κB) signaling pathways [40]. Moreover, in vivo studies have shown a sharp rise in circulating TNF-α levels at the onset of ANFH induced by lipopolysaccharide (LPS) and steroid administration [41]. It remains unclear whether the increase in TNF-α expression observed in ANFH is also applicable to MRONJ, and further accumulation of data is needed. However, the significant increase in TNF-α expression observed in the MRONJ group in the present study is a particularly intriguing finding. Several in vitro studies have investigated the decrease in ALP activity and gene expression to better understand the pathogenesis of MRONJ [29,42]. Furthermore, Peisker et al. reported a significant reduction in ALP levels among MRONJ patients, which supports the findings of the present study [43]. However, some studies have found no clear trend in ALP level changes [44]. As such, the continued accumulation of clinical data will be crucial for reaching more definitive conclusions. In a review of the etiology, pathophysiology, and progression of MRONJ, numerous articles have pointed to an identical suppression of inflammation and bone metabolism, but those studies included both in vitro and in vivo studies, along with clinical investigations [45,46,47,48]. However, at this stage, it remains unclear whether the biomarkers identified in this study are useful for screening, prognosis, or monitoring, including assessment of disease severity. Further research is needed to determine whether ALPL and TNF-α, which showed significant changes in expression levels in this study, could serve as potential biomarkers. Such research should include additional in vitro and in vivo experimental models, as well as studies using blood samples, similar to the present investigation. Notably, the results presented here were derived from a limited number of patient samples. Therefore, the continued accumulation of well-characterized clinical cases will be essential for resolving various unresolved questions related to MRONJ.

This study had several limitations. First, this investigation used a retrospective design and had a limited sample size. Further, the design did not allow measurement of various factors over time. Park et al. prospectively measured several factors over time [37], and future MRONJ studies may need to apply similar methods. To strengthen the validity of the present study, additional research with a greater number of participants will be essential to substantiate the present findings. In the current study, certain results required cautious statistical interpretation., likely due to the limited sample size. Although these factors could not be incorporated into the present study, future investigations should consider incorporating variables such as the duration and dosage of BMAs, history of dental procedures, smoking status, diabetes mellitus, steroid use, renal function, vitamin D levels, and cancer status or treatment, as these have been identified as potential risk factors for the development of MRONJ. Second, the influences of the primary disease (osteoporosis or bone metastasis), type of BMA, route of administration (intravenous, subcutaneous or oral) and duration of BMA administration cannot be excluded. As these factors may play a significant role, they should be carefully considered in future studies.

Although the number of patients with MRONJ has increased in previous years, the disease remains relatively rare. Continued research that also takes into account the above limitations is thus considered essential, and the continuation of these studies will help patients suffering from MRONJ.

## 5. Conclusions

The results of this study suggest that the suppression of bone metabolism related to chronic inflammation may be involved in the etiology, pathophysiology, and progression of MRONJ. These findings could contribute to the future elucidation of the pathogenesis of MRONJ and the development of novel therapies.

## Figures and Tables

**Table 1:** Characteristics of the study participants.

Variable	Non-MRONJ	MRONJ	*p* Value
Patients (*n*)	31	10	
Age (years); mean ± SD (range)	82.1 ± 4.7	81.5 ± 4.7	0.73
Sex (n)			
Male	5	2	0.84
Female	26	8	
Induction for medication (*n*)			
Osteoporosis	30	7	0.06
Bone metastasis	1	3	
Bone-modifying agent received			
Alendronate	9	1	<0.01
Minodronate	13	2	
Ibandronate	6	1	
Risedronate	3	0	
Bisphosphonate (details unknown)	0	1	
Denosumab	0	5	
Route of administration			
Intravenous/subcutaneous	5	6	0.02
Oral	26	4	
Location of MRONJ			
Mandible	—	7	
Maxilla	—	2	
Mandible and maxilla	—	1	
Stage of MRONJ			
I	—	0	
II	—	7	
III	—	3	

**Table 2:** Factor analysis of the non-MRONJ and MRONJ groups.

Non-MRONJ			
	Factor 1	Factor 2	Factor 3
ACP5	0.56	0.52	0.32
ALPL	0.33	0.91	0.23
Leptin		−0.12	−0.32
OPG	0.43	0.18	0.88
OPN	0.27	0.45	0.28
PDGF-BB	0.76	0.17	
RANKL	0.57	0.22	
TNF-α			0.46
**MRONJ**			
	**Factor 1**	**Factor 2**	**Factor 3**
ACP5	0.64		−0.20
ALPL	0.13	−0.27	−0.68
Leptin	−0.11	0.78	0.35
OPG	0.80	0.16	0.58
OPN	0.96	−0.17	
PDGF-BB	−0.32	−0.34	0.14
RANKL		−0.17	0.75
TNF-α		0.82	

**Table 3:** Proportion of variance and principal score on principal component analysis.

Marker	PC1	PC2	PC3	PC4	PC5	PC6
	(35.5%)	(50.8%)	(64.8%)	(76.3%)	(84.6%)	(92.4%)
OPG	−0.47	−0.04	−0.30	−0.09	0.30	−0.14
OPN	−0.38	0.38	−0.07	0.18	0.61	−0.19
PDGF-BB	−0.07	−0.65	0.45	0.003	0.50	0.35
ALPL	−0.47	0.12	0.13	0.13	−0.35	0.42
ACP5	−0.50	0.11	0.15	−0.10	−0.24	0.32
Leptin	0.11	0.42	0.39	−0.78	0.17	0.08
RANKL	−0.33	−0.27	0.42	−0.11	−0.27	−0.74
TNF-α	−0.17	−0.39	−0.58	−0.57	−0.06	0.05

## Data Availability

The data presented in this study are available on request from the corresponding author (the data are not publicly available due to privacy concerns).

## Abbreviations

The following abbreviations are used in this manuscript:

MRONJ	Medication-related osteonecrosis of the jaw
BMAs	Bone-modifying agents
BPs	Bisphosphonates
Dmab	Denosumab
CTX	C-terminal telopeptide
OPG	Osteoprotegerin
OPN	Osteopontin
PDGF-BB	Platelet-derived growth factor BB
ALPL	Alkaline phosphatase liver/bone/kidney
ACP5	Acid phosphatase 5, tartrate-resistant
RANKL	Receptor activator of nuclear factor-κB ligand
TNF-α	Tumor necrosis factor-α
IL-6	Interleukin 6
PTH	Parathyroid hormone
BMP-2	Bone morphogenetic protein 2
DKK-1	Dickkopf WNT signaling pathway inhibitor 1
PCA	Principal component analysis
25(OH)D	25-hydroxyvitamin D
